# An introduction to the maximum entropy approach and its application to inference problems in biology

**DOI:** 10.1016/j.heliyon.2018.e00596

**Published:** 2018-04-13

**Authors:** Andrea De Martino, Daniele De Martino

**Affiliations:** a Soft & Living Matter Lab, Institute of Nanotechnology (NANOTEC), Consiglio Nazionale delle Ricerche, Rome, Italy; b Italian Institute for Genomic Medicine (IIGM), Turin, Italy; c Institute of Science and Technology Austria, Klosterneuburg, Austria

**Keywords:** Systems biology, Molecular biology, Mathematical bioscience, Computational biology, Bioinformatics

## Abstract

A cornerstone of statistical inference, the maximum entropy framework is being increasingly applied to construct descriptive and predictive models of biological systems, especially complex biological networks, from large experimental data sets. Both its broad applicability and the success it obtained in different contexts hinge upon its conceptual simplicity and mathematical soundness. Here we try to concisely review the basic elements of the maximum entropy principle, starting from the notion of ‘entropy’, and describe its usefulness for the analysis of biological systems. As examples, we focus specifically on the problem of reconstructing gene interaction networks from expression data and on recent work attempting to expand our system-level understanding of bacterial metabolism. Finally, we highlight some extensions and potential limitations of the maximum entropy approach, and point to more recent developments that are likely to play a key role in the upcoming challenges of extracting structures and information from increasingly rich, high-throughput biological data.

## 1 Introduction

It is not unfair to say that the major drivers of biological discovery are currently found in increasingly accurate experimental techniques, now allowing to effectively probe systems over scales ranging from the intracellular environment to single cells to multi-cellular populations, and in increasingly efficient bioinformatic tools, by which intracellular components and their putative interactions can be mapped at genome and metabolome resolution. Yet, at least to some degree, these approaches still appear hard to integrate into quantitive predictive models of cellular behaviour. In a sense this is not surprising. Even if we possessed detailed information about all sub-cellular parts and processes (including intracellular machines, their interaction partners, regulatory pathways, mechanisms controlling the exchange with the medium, etc.), it would be hard to build a comprehensive mechanistic model of a cell, and possibly even harder to infer deep organization principles from it. In large part, this is due to the fact that cells have an enormous number of degrees of freedom (e.g. protein levels, RNA levels, metabolite levels, reaction fluxes, etc.) which, collectively, can take on an intimidatingly large number of physico-chemically viable states. On the other hand, experiments necessarily probe only a tiny portion of these states. Therefore, understanding how all internal variables might coordinate so that certain “macroscopic” quantities, like the cell's growth rate, behave as observed in experiments is quite possibly a hopeless task. In addition, these models would most likely require some tuning of the multitude of parameters that characterize intracellular affairs, rendering overfitting a very concrete prospect. At the same time, though, the deluge of data coming from both sides (experiments and bioinformatics) begs for the development of bridges connecting them, not just as descriptive frameworks and predictive tools but also as guides for novel targeted experiments and bioengineering applications.

The problem appears to be that of finding a reasonable ‘middle-ground’ between full fledged mechanistic approaches and qualitative phenomenological descriptions based on coarse-grained quantities only. Perhaps following the lesson of thermodynamics (which, one might argue, has faced a similar question of bridging microscopic and macroscopic descriptions of physical systems), an increasing number of studies is undertaking a route different from – and in many ways inverse of – the mechanistic one. The key issues to be faced along such route are the following: To what degree do experimental results constrain the space of allowed states of a living system? Can we learn something about internal variables from experiments that probe a relatively small number of states? Is there a way to perform reliable statistical inference on the values of un-observed internal variables from empirical data? Ultimately, all of these questions boil down to the problem of inferring probability distributions (or, in other terms, statistical models) from limited data. This age-old challenge dating back to the origin of probability theory (see e.g. Laplace's ‘principle of indifference’, Ref. [1], Ch. 2) has found a self-consistent answer, the only such answer under certain conditions, in the so-called *principle of maximum entropy*.

Over the past decade, entropy maximization or closely related ideas have been repeatedly employed for the analysis of large-scale biological data sets in contexts ranging from the determination of macromolecular structures and interactions [2], [3], [4], [5], [6], [7], [8], [9], [10], [11], [12], [13], [14] to the inference of regulatory [15], [16], [17], [18], [19] and signaling networks [20], [21], [22], [23] and of the organization of coding in neural populations [24], [25], [26], [27], [28], [29], [30], [31], [32], [33], [34], [35], [36], [37]; from the analysis of DNA sequences (e.g. for the identification of specific binding sites) [38], [39], [40] to the study of the HIV fitness landscape [41], [42], [43]; from the onset of collective behaviour in large animal groups [44], [45], [46] to the emergence of ecological relationships [47], [48], [49], [50], [51], [52], [53], [54], [55], [56], [57]. The type of insight derived from these models is remarkably diverse, from fundamental organization principles in structured populations to specific gene-gene interaction networks. Revealingly, the challenge of dealing with high dimensional and limited data posed by biology has in turn stimulated the search for novel efficient implementations of the maximum entropy principle at the interface between computational biology, statistical physics and information theory, leading to an impressive improvement of inference schemes and algorithms. Future ramifications of these studies are likely to explore new application areas, as more/better data become available, theoretical predictions get sharper, and computational methods improve. In many ways, the maximum entropy approach now appears to be the most promising provider of ‘middle grounds’ where empirical findings and bioinformatic knowledge can be effectively bridged.

Several excellent reviews, even very recent ones, cover the more technical aspects of maximum entropy inference from the viewpoint of statistical physics, computational biology or information theory (see e.g. [58], [59], [60], [61], [62]). Our goal here is to provide a compact, elementary and self-consistent introduction to entropy maximization and its usefulness for inferring models from large-scale biological data sets, starting from the very basics (i.e. from the notion of ‘entropy’) and ending with a recent application (a maximum entropy view of cellular metabolism). We mainly hope to convey its broad applicability and potential to deliver new biological insight, and to stimulate further cross-talk while keeping mathematics to a minimum. A few basic mathematical details are nevertheless given in the Supplementary Material for sakes of completeness. Given the vastness of the subject, we shall take a shortcut through most of the subtleties that have accompanied the growth of the field since the 1940s, focusing instead on the aspects that (we believe) are of greater immediate relevance to our purposes. A partial and biased list of additional ingredients and new directions will be presented in the Discussion. The interested reader will however find more details and food for thought in the suggested literature.

For our purposes, the path leading to entropy maximization can start from the intuitively obvious idea that, when extracting a statistical model from data, one should avoid introducing biases other than those that are already present in the data, as they would be unwarranted and discretionary. For instance, if we had to model a process with *E* possible outcomes (like the throw of a dice) and had no prior knowledge of it, our best guess for a probability law underlying this process would have to be the uniform distribution, where each outcome occurs with probability 1/E. In essence, the framework of entropy maximization generalizes this intuition to more complex situations and provides a recipe to construct the ‘optimal’ (i.e. least biased) probability distribution compatible with a given set of data-derived constraints. Central to it is, of course, the concept of ‘entropy’.

## 2 Main text

### 2.1 Entropy and entropy maximization: a bird's eye view

The notion of ‘entropy’ as originated in thermodynamics is usually associated to that of ‘disorder’ by saying that the former can be regarded as a measure of the latter. The word ‘disorder’ here essentially means ‘randomness’, ‘absence of patterns’, or something similar. While not incorrect, these words clearly require a more precise specification to be useful at a quantitative level. As we shall see, once the stage is characterized more clearly, the entropy of a system (e.g. of a population of cells) with prescribed values for certain observables (e.g. the rate of growth of the population) quantifies the number of distinct arrangements of its basic degrees of freedom (e.g. the protein levels, RNA levels, metabolic reaction rates, etc. of each cell) that lead to the same values for the constrained observables. The larger this number, the larger the entropy. In this sense, the entropy of a system is really a measure of the microscopic multiplicity (the ‘degeneracy’) underlying its macroscopically observable state. What makes entropy a powerful inference tool is closely connected to this characterization.

To make things more precise, one can consider a classical, highly stylized example. Imagine having *N* identical balls distributed in *K* urns so that $n_{i}$ balls are placed in the *i*-th urn, with i=1,…,K and $n_{1}+\cdots +n_{K}\equiv \sum_{i=1}^{K}n_{i}=N$. (To fix ideas, one can think of the *N* balls as the *N* different cells and of the *K* urns as *K* distinct configurations for the basic degrees of freedom of each cell. An arrangement $\left\{n_{i}\right\}$ defined by specific values of $n_{1},\ldots ,n_{K}$ therefore represents how cells are distributed over the allowed internal states, with $n_{1}$ cells in state 1, $n_{2}$ in state 2, and so on.) By simple combinatorics (see [63], Ch. 3), the number of ways in which the *N* balls can be placed in the *K* urns leading to the same values of $n_{1},\ldots ,n_{K}$ is given by the multinomial coefficient

(1) $$\Omega =\frac{N!}{n_{1}!n_{2}!\cdots n_{K}!}\;\;.$$

This number^1^ describes the ‘microscopic’ degeneracy underlying the specific arrangement of balls described by the numbers $\left\{n_{1},\ldots ,n_{K}\right\}$ and grows fast with *N*. Actually, for sufficiently large *N*, (1) turns out to be well approximated (see Supplementary Material, Sec. S1) by

(2) $$\Omega ≃e^{NH}\;\;,$$

where

(3) $$H=-\sum_{i=1}^{K}\frac{n_{i}}{N}\ln\frac{n_{i}}{N}\;\;.$$

The quantity *H* defined in (3) is the *entropy* of the arrangement described by the urn occupation numbers $n_{1},\ldots ,n_{K}$. Note (see Supplementary Material, Sec. S2) that H≥0. In a nutshell, Equations (2) and (3) say that, for large *N*, some arrangements $\left\{n_{1},\ldots ,n_{K}\right\}$ can be realized in a huge number of ways (as a matter of fact, in a number of microscopic ways that is exponentially large in *N*), and that the entropy *H* ultimately quantifies this number. On the other hand, some specific arrangements can have a very small degeneracy. For instance, the arrangement with *N* balls in urn 1 and no balls elsewhere (i.e. with $n_{1}=N$ and $n_{i}=0$ for i≠1) can be realized in a unique way, having H=0 and Ω=1.^2^

Note that the quantity $n_{i}/N\equiv p_{i}$ represents the fraction of balls appearing in urn *i* in arrangement $\left\{n_{1},\ldots ,n_{K}\right\}$ or, equivalently for our purposes, the probability that a ball selected at random and uniformly comes from the *i*-th urn. Therefore *H* is a function of the probabilities $\left\{p_{i}\right\}$ (i=1,…,K), i.e.

(4) $$H\equiv H[\{p_{i}\}]=-\sum_{i=1}^{K}p_{i}\ln p_{i}\;\;,$$

and the condition $\sum_{i=1}^{K}n_{i}=N$ simply corresponds to the fact that probabilities should sum to one, i.e. $\sum_{i=1}^{K}p_{i}=1$.

It is rather intuitive that, if balls were ‘thrown’ into urns randomly (i.e. so that each of the *N* balls has equal probability of ending up in any of the *K* urns), the resulting arrangement $\left\{n_{1},\ldots ,n_{K}\right\}$ would much more likely be one with large Ω (and entropy) than one with small Ω (and entropy). In particular, the most likely arrangement $\left\{n_{1}^{⋆},\ldots ,n_{K}^{⋆}\right\}$ (or, equivalently, the most likely distribution of probabilities $\left\{p_{1}^{⋆},\ldots ,p_{K}^{⋆}\right\}$) should coincide with that carrying the largest degeneracy, or maximum entropy, satisfying the constraint $\sum_{i=1}^{N}n_{i}^{⋆}=N$ (or, equivalently, $\sum_{i=1}^{K}p_{i}^{⋆}=1$). In this sense, the safest bet on the outcome of an experiment in which *N* balls are randomly assigned to *K* urns would be to place money on the maximum entropy (MaxEnt) distribution.

This is the gist of the maximum entropy principle: if one is to infer a probability distribution given certain constraints, out of all distributions $\left\{p_{i}\right\}$ compatible with them, one should pick the distribution $\left\{p_{i}^{⋆}\right\}$ having the largest value of (4). The only constraint considered in the above example of balls and urns is the normalization of probabilities, i.e. the fractions $p_{i}$ should sum to one: $\sum_{i=1}^{K}p_{i}=1$. In this case, the MaxEnt distribution is uniform, namely $p_{i}^{⋆}=1/K$ for each i=1,…,K (see Supplementary Material, Sec. S3). However, constraints can involve other quantities, leading to different MaxEnt distributions (see Supplementary Material, Sec. S4 for a few simple examples). This just reflects the diverse information that constraints inject into the inference problem in each case.

It is important to understand that, because they correspond to maximal underlying degeneracy, MaxEnt distributions are the least biased given the constraints: any other distribution compatible with the same constraints would have smaller degeneracy and therefore would artificially exclude some viable (i.e. constraint-satisfying) configurations of the underlying variables. In other terms, a MaxEnt distribution is completely undetermined by features that do not appear explicitly in the constraints subject to which it has been computed.

These ideas, which ultimately make the maximum entropy principle the central conceptual tool for inferring probability distributions subject to constraints, have been placed on firmer and firmer mathematical ground starting from the 1940s. In our view, three classical results are especially noteworthy in the present context.

Firstly, landmark work by Shannon [64] and Khinchin [65] formally characterized *H*, Equation (4), as the *only* function complying with a set of *a priori* requirements (known as Shannon–Khinchin axioms) to be satisfied by a measure of the ‘uncertainty’ or ‘lack of information’ associated to a probability distribution $\left\{p_{i}\right\}$. Here, ‘uncertainty’ relates in essence to how (im)precisely one can identify the configuration of basic degrees of freedom from knowledge of the distribution $\left\{p_{i}\right\}$. In this sense, a larger uncertainty corresponds to a larger underlying degeneracy Ω and hence to a larger entropy *H*. Therefore, MaxEnt distributions compatible with given constraints formally correspond to those that maximize the uncertainty on every feature except for those that are directly encoded in the constraints.

A strict characterization of MaxEnt distributions is instead encoded in a result known as the ‘entropy concentration theorem’ [61]. In short, and referring to the urn-and-balls example discussed above, it rigorously quantifies the observation that, when *N* is sufficiently large, the number of microscopic states underlying the MaxEnt distribution is exponentially larger (in *N*) than the number of microscopic states underlying any other distribution. This is also seen from (2), albeit at a heuristic level. Denoting respectively by $\Omega^{⋆}$ and $H^{⋆}$ the degeneracy and the entropy of the MaxEnt distribution, one sees that, for sufficiently large *N*,

(5) $$\frac{\Omega^{⋆}}{\Omega }≃e^{N(H^{⋆}-H)}$$

for any Ω and *H* corresponding to a distribution different from the MaxEnt one. Because $H^{⋆}$ is the maximum value attained by the entropy, $H^{⋆}-H>0$. Hence, (5) states that microscopic arrangements underlying the MaxEnt distribution are more numerous than those underlying any other distribution by an exponentially large (in *N*) factor. In turn, for large enough *N*, observing an arrangement of balls that corresponds to a distribution different from the MaxEnt one is exponentially (in *N*) less likely.

Finally (and perhaps most importantly for our purposes), *H* has been shown to be the *only* quantity whose constrained maximization allows for least-biased inference satisfying certain generic logical requirements (known as Shore–Johnson axioms) [66]. This result ultimately provides a rigorous basis for using the maximum entropy principle as a general inference technique, independently of the meaning assigned to Eq. (4). In other words, by maximizing *H* one is not looking for a state of maximum indeterminacy (apart from constraints), but rather following the only recipe for self-consistent inference having certain desirable properties. In this sense, the maximum entropy principle ‘simply’ allows to infer least-biased, constraint-satisfying probability distributions in a mathematically rigorous and logically sound manner.

It is clear at this point that the nature of the microscopic variables and the constraints one wants to impose are crucial in the business of using maximum entropy inference in general, and specifically to obtain information about biological systems from complex, high-dimensional data. In addition, the concrete usefulness of MaxEnt distributions besides their theoretical appeal is not *a priori* obvious. Mathematical arguments guarantee that they can provide a compact statistical description of a dataset that is least-biased and compatible with empirical observations. But can that description be employed e.g. for predictive purposes?

### 2.2 Maximum entropy inference in biology: the case of gene interaction networks

The answers to the questions posed above clearly depend on the specifics of the system under consideration and of the available data, and require a case-by-case discussion. Applications of the maximum entropy framework to biology have however become more numerous as the size and quality of data sets has increased, and currently range from protein science and neuroscience to collective animal behaviour and ecology. Such an impressive span suggests that at least some aspects must be recurrent across many if not all of these instances (see the discussion presented in [67] for a broader perspective). It is on these and on the lessons that can be drawn from them that we shall try to focus now. For sakes of clarity and simplicity, we shall phrase things in the context of the study of gene expression. Similar considerations can however be formulated in almost all of the cases listed above.

We begin by re-considering the urn-and-balls model described above in the gene expression scenario. The *N* balls would now represent *N* cellular samples whose complete expression profiles (e.g. RNA levels) have been experimentally characterized, while the *K* urns would represent all possible expression profiles. An expression profile of *R* genes is described by a vector $x=\{x_{i}\}$, where $x_{i}$ stands for the expression level of gene *i*, with i=1,…,R. Usually, the expression of a large set of genes is monitored (R≫1). A specification of an expression profile for each of the *N* cells corresponds to an assignment of the *N* balls to the *K* urns, and fully describes our experimental sample. Note that the number of possible vectors **x**, corresponding to the number *K* of urns, is in principle huge. By contrast, the number of samples (i.e. *N*) is typically much smaller than *K*, so that experiments will vastly under-sample the space of possible expression profiles.

Given the data (i.e. the measured expression profiles), the problem is that of inferring a probability distribution p(x) of expression profiles that is (i) least-biased with respect to unavailable information, and (ii) consistent with empirical constraints. According to the maximum entropy principle, we have to find the distribution p(x) that maximizes the entropy

(6) $$H=-\sum_{x}p(x)\ln p(x)\;\;,$$

subject to data-derived constraints, the above sum being formally carried out over all possible expression profiles. Whether a quantity should be constrained or not is ultimately determined by whether one can reliably estimate it from data or not. In the instances encountered most often and of greater practical relevance, constraints involve low-order moments of the underlying variables, especially averages (first moments) and correlations (second moments). This is because the statistically accurate computation of moments requires more and more experimental samples as the order of the moment increases, so that higher-order moments are generically harder to estimate than lower-order ones. We shall therefore consider only the simplest case in which the mean expression levels of each gene and the gene-gene correlations are required to match those derived from data, namely

(7) $$\bar{x_{i}}=\frac{1}{N}\sum_{a=1}^{N}x_{i,a}\;\;\;\;\;,\;\;\;\;\;\bar{x_{i}x_{j}}=\frac{1}{N}\sum_{a=1}^{N}x_{i,a}x_{j,a}\;\;,$$

where the index *a* runs over samples from 1 to *N*, while *i* and *j* range over genes from 1 to *R*. (It should however be kept in mind that this aspect is ultimately limited by data availability only.) Hence, we must look for the distribution p(x) that maximizes (6) with the usual normalization condition

(8) $$\sum_{x}p(x)=1\;\;,$$

and such that the mean expression levels and the correlations match empirical ones (7), i.e.

(9) $$\sum_{x}x_{i}p(x)=\bar{x_{i}}\;\;\;\text{for all }i=1,\ldots ,R\;,$$

(10) $$\sum_{x}x_{i}x_{j}p(x)=\bar{x_{i}x_{j}}\;\;\;\text{for all }i\text{ and }j\;.$$

Such a distribution $p^{⋆}(x)$ can be computed as shown in Supplementary Material, Sec. S5 (see also [16]) and reads

(11) $$p^{⋆}(x)=\frac{1}{Z}\;e^{\sum_{i=1}^{R}\beta_{i}x_{i}+\sum_{i\leq j}\gamma_{ij}x_{i}x_{j}}\;\;,$$

where *Z*, $\beta_{i}$ (i=1,…,M) and $\gamma_{ij}$ (i,j=1,…,M with i≤j) are constants known as ‘Lagrange multipliers’ that are introduced to enforce the constraints [68]. Equation (11) is often referred to as the ‘pairwise MaxEnt probability distribution’ [24], [28], [40], [62], as it involves at most couplings between pairs of variables through the last term in the argument of the exponential. Clearly, this is due to the fact that only moments up to the second are constrained.

Eq. (11) provides a formal solution to our problem. To fully evaluate it, though, the values of the Lagrange multipliers *Z*, $\beta_{i}$ and $\gamma_{ij}$ have to be computed self-consistently from (8), (9) and (10).^3^ As (11) represents, after all, the least-biased data-informed model for the expression profiles, solving this problem amounts to inferring the model's parameters from data. Performing this task in a realistic context with R≫1 genes, which is ultimately the key for the effective implementation of the maximum entropy framework with biological data sets, can be an extremely challenging computational problem. Luckily, a number of mathematically subtle but computationally effective methods have been developed for this goal over the past decade at the interface between statistical physics and computer science. A discussion of these techniques is however beyond our scopes, and excellent and up-to-date overviews can be found e.g. in [62], [69]. We shall henceforth assume that the parameters $\beta_{i}$'s and the $\gamma_{ij}$'s have been computed, and focus on their physical and biological interpretation.

Based on the MaxEnt distribution (11), one sees that $\beta_{i}$ measures the intrinsic propensity of gene *i* to be expressed, as larger (resp. smaller) values of $\beta_{i}$ favor expression profiles **x** with larger (resp. smaller) values of $x_{i}$. On the other hand, the $\gamma_{ij}$'s characterize the strength of pairwise gene-gene interactions as well as their character (via their signs: positive for positive interactions, negative for negative ones). Hence the $\gamma_{ij}$'s can in principle yield regulatory information that may be scaled up to the reconstruction of an effective genome-resolution gene-gene interaction network. In [16], for instance, such coefficients were used to infer regulatory interactions in *S. cerevisiae*, after expression profiles were experimentally characterized in cultures at different time points (representing the different samples) via microarrays. Ultimately, knowledge of the $\gamma_{ij}$'s allowed to extract a putative, highly-interconnected gene-gene interaction network that emphasized a few hub regulators (including ribosomal and mitochondrial genes as well as genes involved in TOR signaling) implicating global mechanisms devoted to the coordination of growth and nutrient intake pathways.

**Figure 1:**
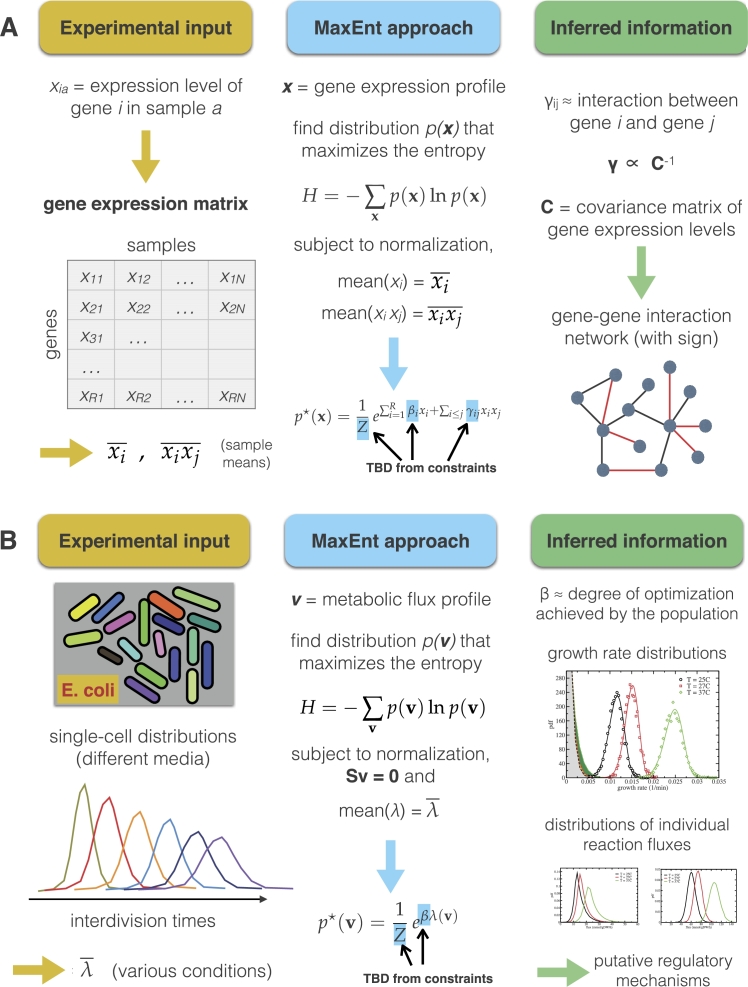
Sketch of the two examples of applications of the maximum entropy principle to biological data analysis discussed in the text. (A) Inference of gene interaction networks from empirical expression data (see Sec. 3 for details). (B) Inference of genome-scale metabolic flux patterns from empirical growth rate distributions in bacteria (see Sec. 4 for details). For each case, we describe schematically the empirical input (left column), the formulation of the maximum entropy inference problem (middle column), and an example of the inferred biological insight (right column).

The ability to bring to light interconnections between genes belonging to different functional categories is a major advantage of the maximum entropy method over alternatives based on the straightforward analysis of correlations, such as clustering techniques. While the latter naturally focus on the identification of genes having a similar expression profile (and therefore tend to group functionally related genes together), the $\gamma_{ij}$'s point to a refined notion of correlation. The origin of this fact is especially transparent when the $x_{i}$'s are taken to be continuous unbounded variables ranging from −∞ to +∞ (a reasonable approximation whenever expression levels are quantified via centered log-fluorescence values). In this case, Eq. (11) describes a multivariate Gaussian distribution and it can be shown that the matrix of $\gamma_{ij}$'s is related to the *inverse* of the matrix of Pearson correlation coefficients, rather than to the correlation matrix itself [16], [62]. This makes a substantial difference. Indeed, the covariance of the expression levels of two genes (say, *A* and *B*) can be large both when *A* and *B* are mutually dependent (e.g. when *A* codes for a transcription factor of *B*) and when, while mutually independent, they both separately correlate with a third gene *C*. In the latter case, though, the behaviour of *C* would explain the observed correlation between *A* and *B*. Specifically, by conditioning on the expression level of *C*, one would see that *A* and *B* are roughly uncorrelated. In other terms, the correlation matrix captures the unconditional correlation between variables and therefore contains effects due to both direct and indirect mechanisms. On the other hand, its inverse describes the correlations that remain once the indirect effects are removed [70], [71], and thereby provides a more robust and consistent characterization of the interactions between variables. (See Figure 1A for a summary of the scenario just discussed.)

Naturally, some adjustments must come in place when one is to go beyond the Gaussian case. Yet, it can be argued that the above picture is quite generic. While the term ‘interaction’ can acquire different meanings in different cases, the MaxEnt distribution focuses on the most relevant part of the correlations and is therefore capable of extracting a more reliable interaction structure from data than that obtainable via a more standard correlation analysis. This property lies in our view at the heart of the success encountered by the maximum entropy method in several applications, in biology as well as in other fields [72], [73], [74]. The above example also shows the centrality of empirical data for maximum entropy inference. In some cases, though, constraints that are not derived from experiments can be employed, together with empirical ones, to guide the inference. An example of this is found in the maximum entropy approach to the analysis of metabolic networks.

### 2.3 Maximum entropy approach to cellular metabolism

Novel experimental techniques employing e.g. microfluidic devices are capable of probing growth in bacterial populations at single cell resolution, yielding detailed data that monitor growth in thousands of individual cells over many generations [75], [76]. These experiments have quantified a number of features linking gene expression and metabolism to overall control mechanisms in proliferating bacteria [77], [78], [79], [80], [81]. While the emergent picture is being increasingly refined, tracking its ‘microscopic’ origin, and particularly the causes of growth rate fluctuations, is largely an open problem. Given the time scales involved in these processes, it is reasonable to think that the regulatory layer controlling energy metabolism is crucially involved in establishing this scenario. Indeed, substantial empirical evidence is connecting growth physiology and heterogeneity to metabolic activity in bacteria [77]. Experimental approaches to characterize the fluxes of intracellular metabolic reactions can provide a population-level picture of the mean activity of central carbon pathways [82]. On the other hand, cellular metabolic networks have been mapped, for many organisms, at the scale of the whole genome [83], and a host of self-consistent computational frameworks exist that can connect metabolic phenotypes (i.e. patterns of material fluxes through the enzyme-catalyzed network of intracellular reactions that processes nutrients into macromolecular building blocks, free energy and biomass) to physiological observables such as the growth rate [84]. The question is whether the reverse problem of relating measured growth rates to flux states of genome-scale networks is feasible. In particular, given the distribution of growth rates found in experiments, can one infer the state of the underlying metabolic network, e.g. the rates of individual reactions? Such knowledge might provide important insight into the metabolic bottlenecks of growth in proliferating cells, which could be especially useful in view of the limited experimental accessibility of intracellular reactions.

To progress along this route, it is important to characterize the map between metabolic networks and growth rates in some more detail. The so-called Constraint-Based Models (CBMs) [85] represent highly effective *in silico* schemes to obtain information about cellular metabolic activity from minimal input ingredients known at genome resolution. To be more specific, the central assumption behind CBMs is that, because of the timescale separation between metabolic (fast) and regulatory (slow) processes, over a sufficiently long time (compared to the cell's cycle) metabolic reactions operate close to a non-equilibrium steady state (NESS) where metabolite and enzyme levels are stationary. Under this homeostatic scenario, a viable configuration of fluxes through enzyme-catalyzed reactions can be represented by a vector $v={\left\{v_{i}\right\}}_{i=1}^{R}$ (with *i* indexing reactions) that satisfies the linear system of equations taking the matricial form

(12) Sv=0,

where **S** denotes the reaction network's matrix of stoichiometric coefficients. From a physical viewpoint, if exchange fluxes with the surrounding medium are included in **S** (as usually done), the above conditions simply express the fact that, at stationarity, the total mass of each chemical species should be conserved, and correspond to Kirchhoff-like laws: the overall production (including external supply) and consumption (including excretions) fluxes of every metabolite should balance. **S** is obtained from genome-scale reconstructions and has *R* columns (one per reaction, numbering to about 1,200 in an organism like *E. coli*) and *C* rows (one per chemical species, amounting to several hundreds for *E. coli*). Therefore (12) compactly represents *C* linear equations with *R* unknowns (the individual fluxes $v_{i}$). Any flux vector **v** solving (12) corresponds in principle to a viable NESS flux pattern of the metabolic network specified by **S**, and the structure of stoichiometric matrices usually allows for an infinite number of solutions. In particular, when ranges of variability of the type $v_{i,\min}\leq v_{i}\leq v_{i,\max}$ are specified for each flux, reflecting empirical kinetic, thermodynamic or regulatory priors (see e.g. [86] for an overview of these data-driven factors), solutions of (12) form a particular kind of set called a ‘convex polytope’ in mathematical jargon [87]. We shall denote convex polytopes by the letter P. On the other hand, to each of the solutions, i.e. to each point in P, CBMs associate a unique value for the biomass output (the growth rate), which we denote as λ(v). Therefore, any rule to sample points from P (i.e. to generate solutions of (12)) will in turn yield a distribution of values for the growth rate. Usually, though, convex polytopes corresponding to genome-scale stoichiometric matrices **S** have very high dimensionality (e.g. several hundreds for *E. coli*), and therefore sadly tend to escape both imagination and computational analysis. We will not detail here how points from P can be generated. For our purposes, it will suffice to say that efficient algorithms exist that allow to extract solutions of (12) with any desired probability distribution for any metabolic network reconstruction, i.e. any **S** (see e.g. [84], [88], [89]).

Given this setup allowing to link metabolic phenotypes to their corresponding growth rates, we are interested in characterizing the inverse map. Specifically, what can we learn about flux configurations from empirical growth rate distributions? Following the maximum entropy idea, one can start by studying the least-biased distribution compatible with data. The simplest data-borne constraint one can impose concerns the mean growth rate. In addition, however, one can inject extra information by requiring that flux vectors are viable NESS of the metabolic network, i.e. that they belong to the polytope of solutions of (12) for the organism under study. This is a substantial change compared e.g. to the case of regulatory networks discussed in the previous section. There, no restriction on the vectors **x** of expression profiles applied as no specific assumption on the ranges of variability and mutual dependence of individual expression levels was made. Here, instead, we are effectively adding a (reasonable and motivated) guess for the underlying model for flux profiles: they should satisfy (12) with pre-determined ranges of variability on each flux. The MaxEnt flux distribution for this case can be computed in full analogy with the previous ones (see Supplementary Material, Sec. S6), and it turns out to be given by

(13) $$p^{⋆}(v)=\left\{ \begin{array}{cc} \frac{1}{Z}\;e^{\beta \lambda (v)} & \text{if }\mathrm{Sv}=0\;\;,\\ 0 & \text{otherwise}\;\;, \end{array}\right.$$

where *Z* and *β* are the Lagrange multipliers respectively ensuring normalization ($\sum_{v}p^{⋆}(v)=1$) and the given mean growth rate. The MaxEnt distribution is only defined on the feasible space P where Sv=0 (i.e. flux vectors must be viable). In addition, the probability to observe a certain **v** depends on its growth rate λ(v), while the parameter *β* quantifies the inferred “degree of optimality”. In a nutshell, a sufficiently large value of *β* causes $p^{⋆}$ to concentrate around flux vectors yielding large values of the growth rate *λ*. On the other hand, as *β* decreases towards more and more negative values, the MaxEnt distribution selects metabolic phenotypes growing more and more slowly. For β=0, $p^{⋆}$ becomes the uniform distribution over the polytope, in which case each viable flux vector **v** is assumed to be equally probable.

**Figure 2:**
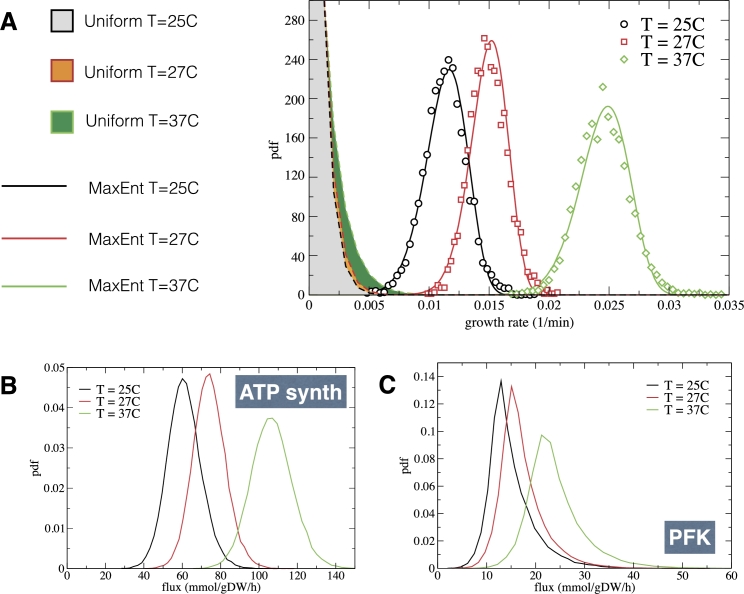
Maximum entropy modeling of growth rate distributions describing *E. coli* growth at different temperatures. Data are taken from [76]. (A) Empirical distributions (markers) are shown together with the MaxEnt distributions obtained by fitting *β* to match the corresponding means (continuous lines), for three different temperatures. For comparison, the dashed lines and the corresponding shaded areas describe the growth rate distributions corresponding to uniform samplings of the solution spaces of the metabolic model, Eq. (12), in the three cases. In each case, such distributions are described by Eq. (14), with *a* = 0, *b* = 22 and different values of *λ*_max_. (B) and (C): inferred distributions of the ATP synthase flux (B) and of the flux through phosphofructokinase (PFK) (C) at different temperatures.

When applied to modeling data describing steady growth of *E. coli* populations using the genome-scale metabolic network reconstruction given in [90], Eq. (13) turned out to reproduce not just the mean growth rates obtained in different experiments for a number of growth medium/strain combinations, but the entire distributions [91]. The fact that the exponential form (13), which ultimately depends on the single parameter *β* (see Supplementary Material, Sec. S6), coincides with empirical distributions confirms the observation that growth rate distributions are one-parameter functions (i.e., the variance is a function of the mean, at odds with Gaussian distributions where the mean and the variance are separate parameters) [79]. Strikingly, recent work has shown that the maximum entropy approach, besides capturing the statistics of the growth rate, is also capable of describing the behaviour of intracellular fluxes belonging to the central carbon processing pathways, which can be estimated by mass spectrometry, without additional assumptions [92]. Equation (13) is likewise capable of describing bacterial growth distributions obtained in a fixed medium at different temperatures (see Figure 2A), while two examples of predictions for how metabolic flux distributions will be modulated as the growth temperature increases are displayed in Figures 2B and 2C. (See Figures 1B for an overview of this case.)

An especially important feature that the maximum entropy approach brings to light is the fact that the value of *β* that provides the best fit to experiments corresponds to mean growth rates that are significantly smaller (usually between 50% and 80%) than the maximum growth rate achievable in the same growth medium according to the CBM prediction, which we denote as $\lambda_{\max}$. In other terms, based on the experimental data sets considered in [76], [91], bacteria appear unable to strictly maximize their growth rates. On the other hand, the distribution of growth rates implied by (13) at β=0 (i.e., when all flux patterns satisfying (12) are equally likely) is of the form

(14) $$q(\lambda )=A\lambda^{a}{\left(\lambda_{\max}-\lambda \right)}^{b}\;\;,$$

where *a* and *b* are constants that depend on the metabolic network reconstruction one is employing^4^ and *A* is a normalization constant. The growth-rate landscape depicted by (14) is extremely heterogeneous, with a small set of states with growth rates close to $\lambda_{\max}$ living in a huge sea of slow-growing flux patterns (see the dashed curves in Figures 2A). This suggests that any random noise added to a fast-growing flux pattern will overwhelmingly likely causes a drastic growth rate reduction.

Taking these observations together, one sees that the space of allowed flux patterns can affect, in a quantitatively measurable way, the growth rate distributions that a population of cells in a given medium will achieve. In particular, one could argue that empirical growth rate distributions found in experiments at single-cell resolution might be explained in terms of a trade-off between the higher fitness of fast-growing phenotypes and the higher entropy (numerosity) of slow-growing ones that is established due to the action of noise. This idea has been tested in a mathematical model of an *E. coli* population evolving in time in the CBM-based fitness landscape (14). Indeed, when bacterial growth rates, along with driving replication, were assumed to fluctuate in time according a small diffusive noise in the feasible space (which contrasts, with high probability, the tendency of the population to concentrate around the fastest growth rates achievable), a scenario that is essentially identical to that described by the MaxEnt distribution was recovered despite starting from very different premises [91]. A more careful mathematical study of the same dynamical model has predicted, among other things, that, within such a scenario, response times to perturbations should be inversely correlated to the difference between the maximum achievable growth rate and the population average [93]. In other terms, populations growing sub-optimally may be more efficient in responding to stresses, a prediction that in principle can be tested experimentally.

Given that a single number, i.e. *β*, appears to provide a full description of empirical growth rate distributions via (13), it would be important to have a more thorough understanding of its physical and biological meaning. According to (13), a larger *β* implies a faster mean growth rate, but can one point to the physico-chemical and biological determinants of growth that contribute to establishing its value in a bacterial population? More specifically, can one identify the factors that limit *β*? In principle, one would expect the growth medium to play a central role in the answer to these questions. However, the picture emerging from a mathematical model in which *β* can be computed from first principles is more involved. In particular, Ref. [94] considered a population growing in a certain environment and characterized by a growth rate distribution r(λ). Imagine sampling $N_{0}$ individuals from that population, each carrying its intrinsic growth rate sampled from r(λ), and subsequently planting them as the initial inoculum in a new growth medium with carrying capacity *K*. By assuming that growth follows the basic logistic model, it was found that the $N_{0}$ seeds evolve in time into a population with growth rate distribution proportional to exp⁡(βλ), where *β* is now a quantity that depends in a mathematically precise way on the capacity-to-inoculum ratio $K/N_{0}$ and on the growth rate distribution from which the seeds were sampled, i.e. on r(λ). The former dependence encodes, as expected, for the growth medium via *K*. Interestingly, though, the presence of $N_{0}$ says that the population maintains a memory of initial conditions. On the other hand, the fact that *β* is also a function of r(λ) points, perhaps unexpectedly, towards history-dependence. This theoretical picture, which characterizes the maximum entropy scenario at a deeper level and provides quantitative support to some possibly intuitive facts (such as history- and inoculum-dependence of growth properties), has been in part confirmed by empirical data on cancer growth rates [94]. Clearly, this oversimplified model does not account explicitly for factors like direct cell-cell interactions or feedbacks between growth and regulation or nutrient availability. Still, it is interesting that the parameter *β*, which in principle is introduced here only to enforce a constraint in the maximum entropy scenario, can be given a well defined physical interpretation. The integration of further empirical data and possibly constraints (e.g. concerning individual fluxes) will hopefully provide new insight into this picture. Preliminary results obtained in this direction are encouraging [95].

While the maximum entropy idea has been employed within CBMs for specific purposes like objective function reconstruction, metabolic pathway analysis or to compute distributions of individual fluxes or chemical potentials over the polytope [96], [97], [98], [99], [100], [101], [102], [103], [104], [105], [106], [107], [108], [109], the approach just discussed presents an overall view of cellular metabolism that differs significantly from that of mechanistic CBMs such as Flux Balance Analysis [110] or related ideas [111], [112], [113], despite the fact that both rely on essentially the same physical NESS assumption via (12). In many ways, the two frameworks appear to be complementary. Flux Balance Analysis is capable of describing the optimal metabolic states of fastest growth achievable by a cell in a multitude of environmental and intracellular conditions. The maximum entropy approach, instead, can clarify in quantitative terms how far from the optimum an actual population is, and what population-level feature might be shaping the observed growth rate heterogeneity. These views might become more tightly linked upon further investigating the underlying regulatory mechanisms, molecular interactions or trade-offs limiting growth.

### 2.4 Beyond the basic framework

The major advantage of the maximum entropy principle lies perhaps in its ability to cope effectively with limited data. The space of states accessible to a living system is huge and always undersampled in experiments. Still, as long as the available data permit the estimation of basic statistical observables with sufficient accuracy, a variety of efficient computational methods exist that allow to compute the parameters of the MaxEnt distribution reliably, leading to a compact, least-biased and mathematically sound representation of a complex, high-dimensional interacting system. We have attempted to describe the fundamentals of entropy maximization and its use for biological applications, opting to focus on two of perhaps the simplest instances involving the study of biological genome-scale networks. Different applications do not require conceptual changes to the approach we presented, but may rely on a more careful and/or involved definition of the state variables (expression profiles or metabolic flux profiles in the examples we considered). Yet some of the issues that we have chosen to leave aside so far now deserve a deeper (albeit brief) discussion.

In first place, we have seen that computing the MaxEnt distribution is akin to constructing a statistical model of the system one is interested in. The inferred model will inevitably depend on the empirical information encoded in the constraints under which entropy is maximized. In turn, while MaxEnt models necessarily reproduce the information used to build them, their predictive power will depend on the encoded constraints as well. In many cases, maximum entropy models can correctly reproduce correlations of order higher than those included as constraints (see e.g. [16], [24] for examples, and [114] for a broader theoretical analysis). Still, predictions concerning other quantities may turn out to be incorrect. Assuming the imposed constraints are factual, this can happen essentially for a unique reason: the constrained quantities do not, by themselves, localize the distribution over states where the new observations are matched. In other terms, more (or different) constraints are required. Clearly, as constraints map to physical or biological ingredients, missing constraints can point to useful insight about of the system under study. An example of this is shown in [115], where some phenomenological aspects of the coexistence between fast-growing and persister phenotypes in bacterial populations are explained in terms of a maximum entropy approach with a constraint on growth rate fluctuations.

Secondly, in our presentation we have taken for granted that cells are indistinguishable (i.e., the arrangement $\left\{n_{1},\ldots ,n_{K}\right\}$ is indistinguishable from the arrangement obtained by exchanging the positions of two balls) and that the set of allowed states is discrete (e.g., *K* urns). As it turns out, these are the simplest and ideal conditions for applying the maximum entropy principle. In many of the biological applications discussed in the literature, it can be argued that both conditions are satisfied. However, this is not generally so. For instance, reaction fluxes are continuous variables and the space of allowed flux configurations is therefore continuous rather than discrete. While nothing invalidates the maximum entropy principle in such cases and the conclusions are unchanged, some more care is needed in setting the stage for it in presence of continuous variables. The interested reader will find more details e.g. in [61], [116].

Thirdly, MaxEnt models like that described by (11) implicitly postulate, via the constraint (10), that the coefficients $\gamma_{ij}$ are symmetric, i.e. $\gamma_{ij}=\gamma_{ji}$. This amounts to assuming that the gene-gene interaction network is *a priori* undirected, which obviously is at odds with a host of biological evidence. To overcome this limitation, which evidently occurs in the maximum entropy approach whenever correlations are constrained, one has to abandon the framework discussed here and resort to methods that infer dynamical models (as opposed to static ones that can be fully described by the probability to observe a certain state). The reasons are rooted in the fundamental distinction between equilibrium and off-equilibrium processes, a nice discussion of which can be found in [60]. Efficient computational methods have been developed to deal with such situations as well, and we again refer the reader to [69] for a recent overview. A specific generalization of the maximum entropy idea that focuses on inferring distributions of *dynamical trajectories* in the configuration space is known as ‘Maximum Caliber’ [117] (see also [118]). Applications of these ideas are presented e.g. in [119], [120], [121], [122], [123] (see also [60] for a review).

Fourth, in our presentation we have tacitly assumed that (i) a MaxEnt distribution exists (i.e., that, once the mathematical problem is constructed, there is a distribution that actually maximizes the entropy subject to the imposed constraints), and that (ii) it is unique (as intuitively desirable to avoid ambiguities, and in compliance with one of the Shore–Johnson axioms). Whether this is the case, ultimately depends on the imposed constraints. For our purposes, it should suffice to say that whenever the constraints are linear functions of the probability distribution $\left\{p_{i}\right\}$, as are e.g. (8), (9) and (10), as well as in all instances discussed here, both (i) and (ii) are true. Yet, in certain situations this may not be the case [124]. The maximum entropy approach then loses some of its appeal and the problem of performing robust inference from limited data has to be treated on a case-by-case basis with extra care.

A final important point we have so far not addressed concerns the treatment of measurement errors affecting the features used to constrain entropy maximization. In practice, inference is always performed under uncertain constraints, which in turn leads to uncertain estimates of the inferred parameters (i.e., for instance, of the inferred interaction structure). The straightforward application of the Maximum Entropy idea discussed so far effectively ignores this aspect. In order to account for it, one can extend the MaxEnt framework presented here in the direction of Bayesian inference, which explicitly deals with probability distributions of parameters given the data [125]. The reconciliation of the Maximum Entropy and Bayesian approaches poses several challenges and has a long and intriguing history [126], [127], up to very recent applications [6], [11], [128], [129], [130]. A related aspect concerns MaxEnt inference with missing or incomplete data, a well-known example of which is discussed in [48], [131]. Such cases have been long considered in the inference literature [132] and specific methods have been developed to deal with them [133], [134], [135]. As more high-resolution data probing large-scale biological networks become available, these techniques are highly likely to be extended well beyond their current application domains.

## 3 Conclusions

The existence of reproducible quantitative relationships connecting the growth of a bacterial population to the composition of the underlying cells (e.g. in terms of the RNA/protein ratio) suggests that, when integrated over large enough cell populations, regulatory and metabolic mechanisms can generate stable outcomes in spite of the heterogeneity and noise that affect them all [136]. Significant deviations from the expected outcome are rare. From a statistical viewpoint, one might say that in such cases the law of large numbers (or more precisely the central limit theorem [137]) is at work, and population-level properties will be roughly independent of the way in which cells in the population distribute over allowed states (or, equivalently, that all viable distributions lead to the same population-level properties). On the other hand, this distribution encodes for critical biological information related to robustness, selection and evolvability, and having access to it would be of paramount importance.

With detailed information about individual intracellular processes, one may hope to construct sufficiently comprehensive mechanistic models capable of mapping the behaviour of individual cells, in terms of their regulatory and metabolic activity, to population-level observables. It is however unclear whether such a ‘direct’ approach, whose concrete realization would be severely hampered by the huge number of variables and parameters to be accounted for, would allow to fully uncover how cells are distributed over allowed regulatory states, as the space of states to be explored would be dauntingly large. The ‘inverse’ route consists in trying to infer the distribution from the observed population-level behaviour. As many distributions are likely to be compatible with empirical results, one would ideally want to be able to sample all of them. The space of such distributions can however be prohibitively large for systems as complex as cells. Therefore, the problem of selecting, out of this space, the most informative distribution has to be faced. The maximum entropy principle provides a constructive and mathematically controlled answer: it is the distribution that maximizes the entropy (4) subject to empirical constraints that yields the optimal choice.

Some of the issues raised above however suggest that, as much as the maximum entropy principle provides powerful means to extract models and/or useful low-dimensional representations from complex, high-dimensional and limited data, there is room to dissect its fundamentals [138], [139], re-analyze its use [140], [141], or search for alternatives [142]. In our view, besides providing essential theoretical insight, these contributions also highlight some of the main practical challenges that biological datasets pose to computational and theoretical scientists, whose ultimate goals are interpreting them and using them to build e.g. predictive models and *de novo* design protocols. These challenges have ultimately been the key driver behind the massive progress achieved in the study of the so-called ‘inverse problems’ in statistical physics over the past decade. As one can only envision that data will continue to get more and more abundant, of higher quality and increasingly diverse (as novel conceptual schemes emerge), the push for technical improvements and new schemes will likely escalate. In turn, maximum entropy methods may spread further in the coming years, as they are capable of extracting the simplest and least biased conclusions that one can reliably draw from limited data. Some of the new directions that are being probed have already shown promise for applications in biology [143], [144].

### Author contribution statement

All authors listed have significantly contributed to the development and the writing of this article.

### Funding statement

This work was supported by the European Union's Horizon 2020 Research and Innovation Staff Exchange program MSCA-RISE-2016 under grant agreement no. 734439 INFERNET, and by the People Programme (Marie Curie Actions) of the European Union's Seventh Framework Programme (FP7/2007-2013) under Research Executive Agency (REA) Grant Agreement No. 291734.

### Competing interest statement

The authors declare no conflict of interest.

### Additional information

Supplementary content related to this article has been published online at https://doi.org/10.1016/j.heliyon.2018.e00596.

No additional information is available for this paper.

## Supplementary material

The following Supplementary material is associated with this article:

Supplementary Material Supplementary Text: basic derivations. Supplementary Material

## Footnotes

1 To give an idea, for N=10 and K=3, the arrangement with $n_{1}=5$, $n_{2}=3$ and $n_{3}=2$ has Ω=2520. If K=4, the arrangement with $n_{1}=5$, $n_{2}=3$ and $n_{3}=n_{4}=1$ has instead Ω=5040 (twice as large). If N=100 and K=3, the arrangement with $n_{1}=50$, $n_{2}=30$ and $n_{3}=20$ has instead $\Omega ≃10^{42}$.

2 Notice however that many arrangements have H=0. In particular, any arrangement with *N* balls in one urn and none in the others has H=0. Hence there are *K* ways to realize an H=0 arrangement, corresponding to the fact that there are *K* possible choices for the urn in which to place the *N* balls.

3 Note that, because $\bar{x_{i}x_{j}}=\bar{x_{j}x_{i}}$, the number of constraints is 1+R+R(R+1)/2 (where the three addends correspond respectively to (8), (9) and (10)), and it matches the number of constants to be determined. This makes the problem of retrieving the values of the above parameters mathematically well defined.

4 For instance, one has a=22 and b=0 for *E. coli*'s on the carbon catabolic core, but a=171 and b=3 for the full genome-scale network given in [90].
