# Supplementary material for: An introduction to the maximum entropy approach and its application to inference problems in biology
Source: Heliyon. 2018 Apr 13;4(4):e00596. doi: 10.1016/j.heliyon.2018.e00596 (PMC5968179; doi:10.1016/j.heliyon.2018.e00596)
Supplement: Supplementary Material — Supplementary Text: basic derivations. [file mmc1.pdf]

An introduction to the Maximum Entropy approach and its application to inference problems in biology

SUPPORTING MATERIAL

Andrea De Martino[1,2,*] and Daniele De Martino[3]

[1]*Soft & Living Matter Lab, Institute of Nanotechnology (NANOTEC), Consiglio Nazionale delle Ricerche, Rome (Italy)*

[2]*Italian Institute for Genomic Medicine (IIGM), Turin (Italy)*

[3]*Institute of Science and Technology Austria, Klosterneuburg (Austria)*

S1. DERIVATION OF H

To evaluate Ω one can apply Stirling's approximation for the factorial, i.e. $m! \simeq m^m e^{-m}$ (valid for sufficiently large m), to each of the terms that appear in

$$\Omega = \frac{N!}{n_1! n_2! \cdots n_K!} \quad . \tag{S1}$$

This yields

$$\Omega \simeq \frac{N^N\, e^{-N}}{n_1^{n_1}\cdots n_K^{n_K}\, e^{-(n_1+\cdots+n_K)}} = \frac{N^N}{n_1^{n_1}\cdots n_K^{n_K}} = e^{\ln(N^N)-\ln(n_1^{n_1}\cdots n_K^{n_K})}$$
$$= e^{N\ln N-\sum_{i=1}^K n_i\ln n_i} = e^{N\left[-\sum_{i=1}^K \frac{n_i}{N}(\ln n_i - \ln N)\right]} = e^{NH} \quad , \tag{S2}$$

where H is the entropy defined in Eq. (3) in the Main Text, i.e.

$$H = -\sum_{i=1}^K \frac{n_i}{N}\ln\frac{n_i}{N} \quad , \tag{S3}$$

and we used (twice) the fact that $n_1 + \cdots + n_K = N$.

S2. $H \geq 0$

Because $\sum_{i=1}^K n_i = N$, the numbers $\{n_i\}$ on which the entropy H (Eq. (S3)) depends are such that $0 \leq n_i \leq N$ (or, equivalently, $0 \leq (n_i/N) \leq 1$) for each i. Therefore, $\ln(n_i/N) \leq 0$ for each i, with the equality holding only when $n_i = N$. In turn, $(n_i/N)\ln(n_i/N) \leq 0$ for each i, with the equality holding only when $n_i = N$ or $n_i = 0$. Hence $H = -\sum_{i=1}^K (n_i/N)\ln(n_i/N) \geq 0$, with $H = 0$ only for the arrangements with $n_i = N$ for some i and $n_j = 0$ for each $j \neq i$.

S3. THE MAXENT DISTRIBUTION ON $\{1,\ldots,K\}$ UNDER A NORMALIZATION CONSTRAINT IS UNIFORM

To find the distribution $\{p_i\}$ that maximizes the entropy as given in Eq. (4) in the Main Text, i.e.

$$H \equiv H[\{p_i\}] = -\sum_{i=1}^K p_i \ln p_i \quad , \tag{S4}$$

* Corresponding author; email: andrea.demartino@roma1.infn.it

subject to the constraint

$$\sum_{i=1}^{K} p_i = 1 \tag{S5}$$

one can resort to the method of Lagrange multipliers [1], which for our purposes amounts to computing the variation (derivative) of the function

$$F = H + \alpha \sum_{i=1}^{K} p_i \tag{S6}$$

over p_i, setting it to zero and isolating p_i from the resulting expression. The first term in (S6) represents the function to be optimized, while the second represents the quantity whose value is constrained (as we are considering a case with the only constrained quantity given in (S5), a single extra term needs to be added to H). The constant α is called a 'Lagrange multiplier', and its value has to be computed self-consistently from the constraint. With some foresight, we write α as $1 - \ln Z$ with Z a constant. Differentiation of F over p_i yields

$$\delta F = \delta \left[-\sum_{i=1}^{N} p_i \ln p_i + (1 - \ln Z) \sum_{i=1}^{K} p_i \right] = \left[-\ln p_i - \ln Z \right] \delta p_i \quad . \tag{S7}$$

The above expression vanishes for $p_i = p_i^\star \equiv 1/Z$, which is the MaxEnt distribution we sought for. It remains to evaluate Z. This is done from the condition $\sum_{i=1}^{K} p_i^\star = 1$, which takes the form $K/Z = 1$. Hence we find $Z = K$, and the MaxEnt distribution is finally given by $p_i^\star = 1/K$ for each $i = 1, \ldots, K$, i.e. by the uniform distribution on $\{1, \ldots, K\}$.

S4. THE MAXENT DISTRIBUTION UNDER DIFFERENT CONSTRAINTS

Constrained mean. If, besides the normalization (S5), one is interested in constraining the mean of a certain variable x that takes values x_i in the K states (with $i = 1, \ldots, K$), one should impose an additional constraint on the quantity

$$\sum_{i=1}^{K} x_i p_i = \overline{x} \quad . \tag{S8}$$

Proceeding as above, the function F now reads

$$F = H + (1 - \ln Z) \sum_{i=1}^{K} p_i + \beta \sum_{i=1}^{K} x_i p_i \quad , \tag{S9}$$

with β the Lagrange multiplier associated to (S8). Differentiation now gives

$$\delta F = \left[-\ln p_i - \ln Z + \beta x_i \right] \delta p_i \quad , \tag{S10}$$

whence the MaxEnt distribution

$$p_i^\star = \frac{1}{Z}\, e^{\beta x_i} \tag{S11}$$

follows. In this case, $p_i^\star$ is exponential. As before, the Lagrange multiplies Z is fixed by the condition $\sum_{i=1}^{K} p_i^\star = 1$ to $Z = \sum_{i=1}^{K} e^{\beta x_i}$. β, on the other hand, should be determined from the requirement that

$$\sum_{i=1}^{K} x_i p_i^\star = \overline{x} \quad . \tag{S12}$$

Constrained mean and second moment. In order to impose, together with (S5) and (S8), an additional constraint on the second moment of x, i.e. on

$$\sum_{i=1}^{K} x_i^2 p_i = \overline{x^2} \quad , \tag{S13}$$

one should differentiate the function

$$F = H + (1 - \ln Z)\sum_{i=1}^{K} p_i + \beta \sum_{i=1}^{K} x_i p_i + \gamma \sum_{i=1}^{K} x_i^2 p_i \quad . \tag{S14}$$

It turns out that the MaxEnt distribution has a Gaussian form, namely

$$p_i^\star = \frac{1}{Z}\, e^{\beta x_i + \gamma x_i^2} \quad , \tag{S15}$$

where the three Lagrange multipliers Z, β and γ have to be determined from the three conditions

$$\sum_{i=1}^{K} p_i^\star = 1 \quad , \quad \sum_{i=1}^{K} x_i p_i^\star = \overline{x} \quad , \quad \sum_{i=1}^{K} x_i^2 p_i^\star = \overline{x^2} \quad . \tag{S16}$$

Constrained mean of the logarithm. Finally, we impose (S5) together with a constraint on the mean value of the logarithm of x, namely

$$\sum_{i=1}^{K} p_i \ln x_i = \overline{\ln x} \quad . \tag{S17}$$

Differentiating

$$F = H + (1 - \ln Z)\sum_{i=1}^{K} p_i + \gamma \sum_{i=1}^{K} p_i \ln x_i \quad , \tag{S18}$$

one finds

$$p_i^\star = \frac{x_i^\gamma}{Z} \quad . \tag{S19}$$

In other terms, the MaxEnt distribution is in this case a power law. Again, the Lagrange multipliers Z and γ have to be determined from the constraints

$$\sum_{i=1}^{K} p_i^\star = 1 \quad , \quad \sum_{i=1}^{K} p_i^\star \ln x_i = \overline{\ln x} \quad . \tag{S20}$$

S5. MAXIMUM ENTROPY INFERENCE IN A COMPLEX SETTING: PAIRWISE MAXENT DISTRIBUTIONS

Based on the examples discussed in the previous section, the least biased distribution $p(\mathbf{x})$ compatible with the empirical constraints is found by differentiating the function

$$F = H + (1 - \ln Z)\sum_{\mathbf{x}} p(\mathbf{x}) + \sum_{i=1}^{R} \beta_i \sum_{\mathbf{x}} x_i p(\mathbf{x}) + \sum_{i\le j} \gamma_{ij} \sum_{\mathbf{x}} x_i x_j p(\mathbf{x}) \tag{S21}$$

with respect to $p(\mathbf{x})$, where H is the entropy defined in Eq. (6) in the Main Text, namely

$$H = -\sum_{\mathbf{x}} p(\mathbf{x}) \ln p(\mathbf{x}) \quad . \tag{S22}$$

Note that we have introduced one Lagrange multiplier for the constraints enforcing normalization (i.e. $\sum_{\mathbf{x}} p(\mathbf{x}) = 1$; this requires one multiplier denoted by Z), mean (i.e. $\sum_{\mathbf{x}} x_i p(\mathbf{x}) = \overline{x_i}$ for all i; this requires one multiplier for each i, hence R in total, denoted by β_i with $i = 1, \ldots, R$) and correlations (i.e. $\sum_{\mathbf{x}} x_i x_j p(\mathbf{x}) = \overline{x_i x_j}$ for all i and j; this requires one multiplier for each pair (i, j) with $i \le j$, hence $R(R+1)/2$ in total denoted by γ_{ij}). This results in the MaxEnt distribution

$$p^\star(\mathbf{x}) = \frac{1}{Z}\, e^{\sum_{i=1}^{R} \beta_i x_i + \sum_{i\le j} \gamma_{ij} x_i x_j} \quad . \tag{S23}$$

Again, the total number of parameters to be determined matches that of constraints, and the values of the $1 + R + R(R+1)/2$ Lagrange multipliers are to be determined from the $1 + R + R(R+1)/2$ conditions

$$\sum_{\mathbf{x}} p^\star(\mathbf{x}) = 1 \quad , \quad \sum_{\mathbf{x}} x_i p^\star(\mathbf{x}) = \overline{x_i} \quad , \quad \sum_{\mathbf{x}} x_i x_j p^\star(\mathbf{x}) = \overline{x_i x_j} \ . \tag{S24}$$

When the x_i?s are continuous variables ranging from $-\infty$ to $+\infty$ one can directly relate Lagrange multipliers to the inverse of the correlation matrix, as shown e.g. in [2]. In this so-called 'mean-field' case, Eq. (S23) is a multivariate Gaussian. In general, though, when R is large and data are taken from experiments, this problem can only be solved *in silico*. As this can prove to be a daunting task, several methods have been developed to achieve an efficient numerical solution (see e.g. [3] for a recent comprehensive review).

S6. ENTROPY MAXIMIZATION IN METABOLIC NETWORKS SUBJECT TO CONSTRAINED MEAN GROWTH RATE

The MaxEnt distribution of flux patterns constrained by the empirical mean growth rate $\overline{\lambda}$ is found by differentiating the function

$$F = H + (1 - \ln Z) \sum_{\mathbf{v}} p(\mathbf{v}) + \beta \sum_{\mathbf{v}} \lambda(\mathbf{v}) p(\mathbf{v}) \ , \tag{S25}$$

where

$$H = -\sum_{\mathbf{v}} p(\mathbf{v}) \ln p(\mathbf{v}) \tag{S26}$$

is the entropy, in full analogy with (S9). One gets

$$p^\star(\mathbf{v}) = \frac{1}{Z} e^{\beta \lambda(\mathbf{v})} \ . \tag{S27}$$

The restriction to the polytope of solutions can be applied straightforwardly by imposing that configurations $\mathbf{v}$ satisfy the mass balance conditions $\mathbf{S}\mathbf{v} = \mathbf{0}$ (viz. Eq. (21) in the Main Text) with the prescribed bounds of variability on each flux, i.e.

$$p^\star(\mathbf{v}) = \begin{cases} \frac{1}{Z} e^{\beta \lambda(\mathbf{v})} & \text{if } \mathbf{S}\mathbf{v} = \mathbf{0} \ , \\ 0 & \text{otherwise} \ . \end{cases} \tag{S28}$$

The values of Z and β have to be determined from the conditions

$$\sum_{\mathbf{v}} p^\star(\mathbf{v}) = 1 \ , \tag{S29}$$

$$\sum_{\mathbf{v}} p^\star(\mathbf{v}) \lambda(\mathbf{v}) = \overline{\lambda} \ . \tag{S30}$$

Alternatively, one can add an extra constraint in (S25) enforcing that, for each $\mathbf{v}$, $\mathbf{S}\mathbf{v} = \mathbf{0}$. The value of Z is automatically set by (S29) to $Z = \sum_{\mathbf{v}} e^{\beta \lambda(\mathbf{v})}$ (where the sum is over flux vectors such that $\mathbf{S}\mathbf{v} = \mathbf{0}$), so that (S28) ultimately depends on a single parameter, i.e. β.

[1] Bertsekas, D.P., 2014. *Constrained optimization and Lagrange multiplier methods* (Academic Press).

[2] Stein, R.R., Marks, D.S. and Sander, C., 2015. Inferring pairwise interactions from biological data using maximum-entropy probability models. *PLoS Comp Biol*, 11(7), e1004182. doi:10.1371/journal.pcbi.1004182

[3] Nguyen, H.C., Zecchina, R. and Berg, J., 2017. Inverse statistical problems: from the inverse Ising problem to data science. *Adv Phys*, 66(3), 197-261. doi:10.1080/00018732.2017.1341604
